# Conformational Control of the Binding of the Transactivation Domain of the MLL Protein and c-Myb to the KIX Domain of CREB

**DOI:** 10.1371/journal.pcbi.1002420

**Published:** 2012-03-15

**Authors:** Elif Nihal Korkmaz, Ruth Nussinov, Türkan Haliloğlu

**Affiliations:** 1 Polymer Research Center & Chemical Engineering Department, Boğaziçi University, Istanbul, Turkey; 2 Basic Science Program, SAIC-Frederick, Inc., Center for Cancer Research Nanobiology Program, NCI-Frederick, Frederick, Maryland, United States of America; 3 Sackler Institute of Molecular Medicine, Department of Human Genetics and Molecular Medicine, Sackler School of Medicine, Tel Aviv University, Tel Aviv, Israel; MRC Laboratory of Molecular Biology, United Kingdom

## Abstract

The KIX domain of CBP is a transcriptional coactivator. Concomitant binding to the activation domain of proto-oncogene protein c-Myb and the transactivation domain of the trithorax group protein mixed lineage leukemia (MLL) transcription factor lead to the biologically active ternary MLL∶KIX∶c-Myb complex which plays a role in Pol II-mediated transcription. The binding of the activation domain of MLL to KIX enhances c-Myb binding. Here we carried out molecular dynamics (MD) simulations for the MLL∶KIX∶c-Myb ternary complex, its binary components and KIX with the goal of providing a mechanistic explanation for the experimental observations. The dynamic behavior revealed that the MLL binding site is allosterically coupled to the c-Myb binding site. MLL binding redistributes the conformational ensemble of KIX, leading to higher populations of states which favor c-Myb binding. The key element in the allosteric communication pathways is the KIX loop, which acts as a control mechanism to enhance subsequent binding events. We tested this conclusion by *in silico* mutations of loop residues in the KIX∶MLL complex and by comparing wild type and mutant dynamics through MD simulations. The loop assumed MLL binding conformation similar to that observed in the KIX∶c-Myb state which disfavors the allosteric network. The coupling with c-Myb binding site faded, abolishing the positive cooperativity observed in the presence of MLL. Our major conclusion is that by eliciting a loop-mediated allosteric switch between the different states following the binding events, transcriptional activation can be regulated. The KIX system presents an example how nature makes use of conformational control in higher level regulation of transcriptional activity and thus cellular events.

## Author Summary

CBP (CREB-binding protein) is a transcriptional regulator of RNA polymerase II-mediated transcription. KIX is a domain of CBP. KIX binding to the activation domain of proto-oncogene protein c-Myb and the transactivation domain of the trithorax group protein mixed lineage leukemia (MLL) transcription factor forms the biologically active ternary MLL∶KIX∶c-Myb complex. This complex has been shown to play a key role in Pol II-mediated transcription. Experimental data show that the binding of the activation domain of MLL to KIX enhances c-Myb binding. Here, we studied the c-Myb∶KIX∶MLL ternary structure and models based on this structure, KIX-only, KIX-MLL, and c-Myb-KIX, by explicit solvent molecular dynamics (MD) simulations. MD simulations can help in figuring out allosteric events and thus functional mechanisms. Our *in silico* analysis of the dynamic behavior indicated that when MLL is bound, there is allosteric communication between the two KIX binding sites. We observed a shift in the conformational ensemble of KIX upon the binding of the MLL activation domain, leading to a pre-organization of the KIX∶c-Myb binding site and explaining the enhanced c-Myb binding observed experimentally. On the other hand, this is not the case if c-Myb binds to KIX, which suggests that KIX regulation is under conformational control.

## Introduction

Allostery plays a crucial role in biological processes on the molecular and cellular levels [1]–[3]. Allostery involves communication between binding sites [4]–[6]. Following a binding event, the most populated conformation may differ from the one which prevails in the free state. This can be described by a shift in the free energy landscape, where the populations in the conformational ensembles are redistributed [1], [7]–[10]. The formation of the ternary complex of the KIX domain of the CREB (cyclic-AMP responsive element binding protein) binding protein (CBP or CREBBP), the transactivation domain of the MLL, and the proto-oncogene protein c-Myb is cooperative. Binding of MLL to KIX elicits an allosteric effect that enhances the binding of KIX to c-Myb [11]. The affinity of c-Myb binding to KIX-MLL binary complex is two-fold higher than of KIX alone [7], [12].

MLL and c-Myb are crucial elements for normal blood cell development. c-Myb is expressed widely in cells in the proliferative state, as in hematopoietic cells, whereas MLL ensures that the transcriptionally active state of a group of genes is maintained. CBPs play roles in cellular growth, differentiation, proliferation and apoptosis [13]–[15]. CBP binds to phosphorylated CREB, and is a modular coactivator of RNA polymerase II-mediated transcription [13], [16], [17]. It has several interacting domains which provide a scaffold for multiprotein assembly, including transcription factors, signaling molecules and hormone receptors [14], [18]–[20]. Thus, the control of the KIX binding states is crucial, and is related to many diseases, from cancer to neurodegenerative diseases [13], [18]–[23]. (See supporting information Text S1).

**Figure 1:**
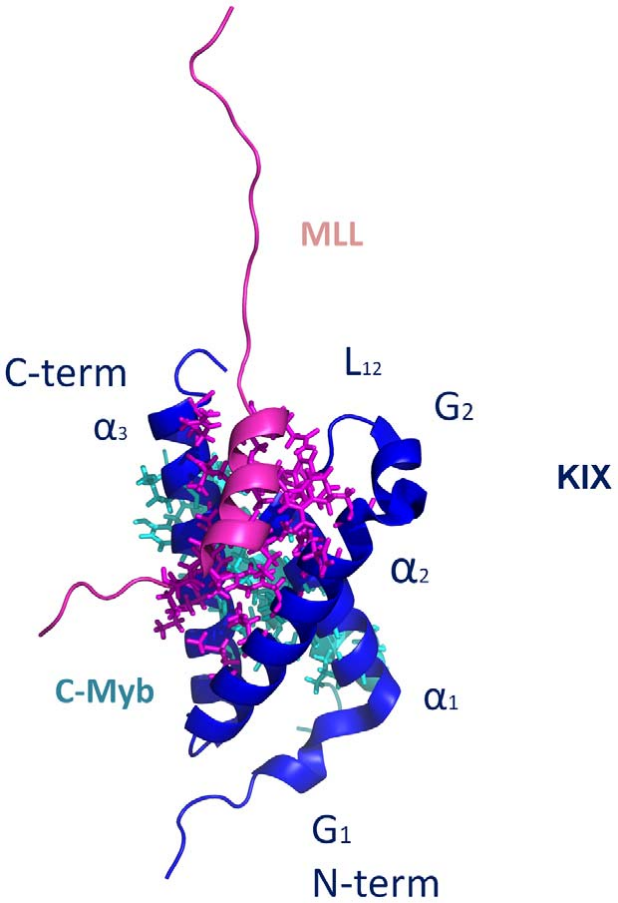
Structure of c-Myb∶KIX∶MLL ternary complex. 3-D Representation of the c-Myb∶KIX∶MLL (PDB id: 2AGH [11]) structure using PyMOL [44]. KIX, MLL and c-Myb structures are shown in purple, pink and cyan respectively. Secondary structure elements are labeled on KIX. The regions shown in sticks representations are the sites on KIX that are in contact with MLL (pink) and c-Myb (cyan).

CBP has two distinct binding surfaces on the KIX domain that can be occupied simultaneously [12]. The activation domains of several proteins including MLL, Tax, Tat and c-Jun can bind these sites which are located roughly opposite to the c-Myb binding site [24], [25]. The KIX structure consists of a three helix bundle (α_1_, residues: Gln597–Ile611; α_2_, residues: Arg623–Tyr640; α_3_, residues: Arg646–Arg669) and a 3_10_ helix (G_1_, residues: Trp591–His594) at the N-terminal. The C-terminal region is linked to a second 3_10_ helix (G_2_, residues Pro617– Lys621) through a loop (L_12_) [11] (Figure 1). The binding of the MLL activation domain occurs on the hydrophobic groove formed by the L_12_ loop and the α_2_ helix region of KIX, specifically by the side chains of Ile611 and Phe612, aliphatic region of Arg624, Leu628, Tyr631 and Leu664. The side chains of Ile2849, Phe2852, Val2853, Leu2854 and Thr2857 (Ile849, Phe852, Val853, Leu854 and Thr857 with the numbering convention in the structure file [11]) of MLL make contacts with Phe612, Pro613, Arg624, Asn627, Leu628 and Tyr631 in the hydrophobic binding groove of KIX. Ile2844, Leu2845 and Pro2846 (Ile844, Leu845 and Pro846) of MLL fit in the hydrophobic binding groove of KIX consisting of residues Met639, Lys656, Ile660, Ile664 and Arg668 (Text S1).

The c-Myb activation domain-binding site resides on the hydrophobic groove which is formed by the first and third helices of KIX, specifically by the side chains of Leu599, His602, Leu603, the aliphatic portion of Lys606, Leu607, Ala610 from the α_1_ helix, Tyr650, Leu653, Ala654, Ile657, Tyr658 and the aliphatic region of Lys662 from the α_3_ helix [11]. This binding groove provides a docking surface for the nonpolar side of c-Myb consisting of the side chains of Ile295, Leu298, Leu301, Leu302, Met303, Thr305 and Leu309. This hydrophobic groove is responsible for more than half of the interactions between c-Myb and KIX. The electrostatic interactions of c-Myb Arg294 with Glu665 of KIX along with the interactions of c-Myb Glu306 and KIX Arg646 determine the orientation of c-Myb in the groove [26]. Stabilization of the C-terminal of the α_3_ helix of KIX, which has most of the allosterically important residues, was suggested to increase the electrostatic interactions by bringing Glu665 and Glu666 of KIX in close proximity to Arg294 and Lys291 of c-Myb, respectively. The two hydrophobic binding regions which c-Myb and MLL bind (Figure 1) have no direct contact [11]. However, binding is cooperative, which is why it is considered allosteric.

Significant changes are observed at the MLL binding interface of KIX in the ternary complex compared to the KIX∶c-Myb binary complex, with the disordered regions in KIX in the KIX∶c-Myb binary structure becoming structured in the ternary structure [11]. Two regions become more ordered: loop L_12_ (between helices α_1_ and G_2_ regions, Figure 1) is observed to be in contact in the ternary complex and the extended C-terminal end (the end of the α_3_ helix) is stabilized in the presence of MLL [11]. Recent findings suggest that formation of the binary KIX-MLL complex leads to a redistribution of the KIX conformational ensemble, allosterically activating the c-Myb binding site, and that the conformation of KIX in the KIX-MLL binary complex resembles that in the ternary complex [7].

In this work, we studied the c-Myb∶KIX∶MLL ternary structure (PDB code: 2AGH [11]) and models based on this structure (described in the Materials and Methods section), by explicit solvent molecular dynamics (MD) simulations. MD simulations can help in figuring out the allosteric events to understand functional mechanisms [2], [7], [27]–[31]. Comparative analysis of the dynamic behavior showed that there is allosteric communication between the two binding sites on KIX when MLL is bound. The shift in the conformational ensemble of KIX upon the binding of the MLL activation domain demonstrated that the binding pre-organizes the KIX∶c-Myb binding site, which explains the more favorable interaction.

## Results/Discussion

### Allostery in c-Myb∶KIX∶MLL

**Figure 2:**
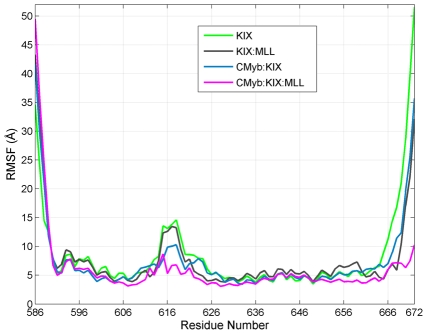
Root Mean Square Fluctuations (RMSF) of KIX from different simulations. The RMSF of KIX residues from the KIX-only (green), KIX∶MLL (grey), c-Myb∶KIX (blue) and c-Myb∶KIX∶MLL (pink) simulations. The high RMSF values around Phe612∶Lys621 (L_12_ and G_2_) of KIX indicate that this region is the most flexible. Comparing C-Myb∶KIX (green) with KIX (blue) and KIX∶MLL (red) cases, it can be concluded that c-Myb binding rigidifies this region.

**Figure 3:**
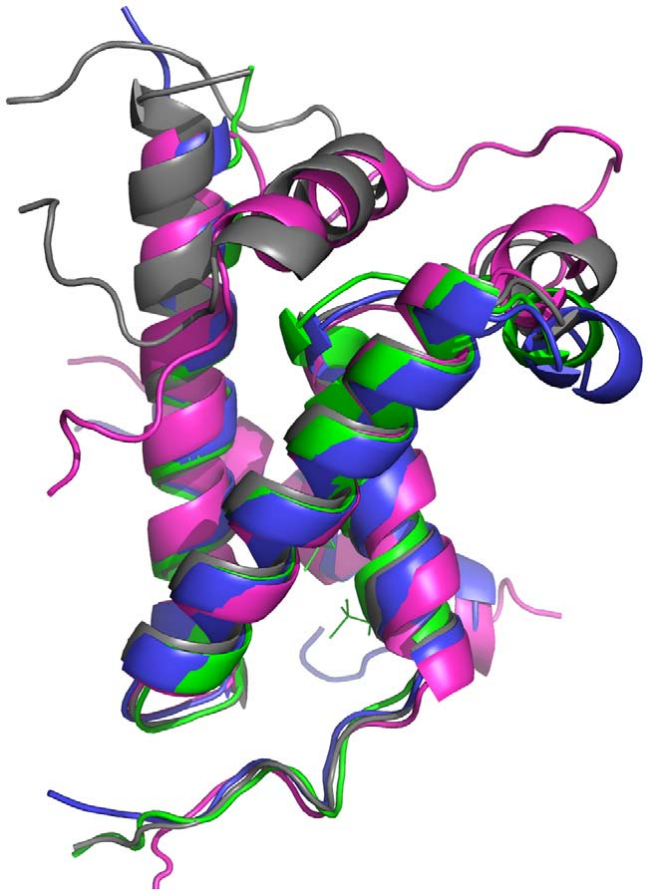
Movement of L_12_-G_2_ loop. The alignment of the average structures from the KIX-only (green), KIX∶MLL (grey), c-Myb∶KIX (blue) and c-Myb∶KIX∶MLL (pink) simulations shown using PyMOL [44].

From the RMSF (root mean square fluctuations) of KIX, KIX∶MLL, c-Myb∶KIX and c-Myb∶KIX∶MLL simulations, the most mobile region of KIX is at Phe612∶Lys621 (Figure 2). This region corresponds to the L_12_ and G_2_ parts of the KIX structure (Figure 1), which is located close to the MLL binding site [11]. Comparing the RMSF of the simulations of the four models, we observed that c-Myb binding reduces the mobility of the L_12_ and G_2_ region. Similar behavior is also observed in parallel simulations (Figure S1). Further, the movement of this region is accompanied by the motion of the Phe612 side chain, which is at the MLL binding site and has a role in signal transmission to the c-Myb binding site (Figure 3). Upon binding of MLL, the side chain of Phe612 fits itself in the core formed by residues Phe2851, Val2852 and Asn2856 (Phe851, Val852 and Asn856) forming hydrophobic contacts with the MLL amphipathic helix [11]. In Figure 3, the motion of the L_12_ and G_2_ region can be observed clearly. Without MLL and c-Myb (i.e. in the KIX-only simulation) this region bends towards the α_1_ helix; in the presence of c-Myb (the c-Myb∶KIX simulation) this region moves further apart restricting the conformational space of the loop region similar to the conformation in the KIX-only simulation (Figure 3). This conformation of the loop might have a role in MLL binding as the conformational space of MLL binding interface becomes more accessible. As the loop gets stabilized in this state, the α3 helix of KIX which is in contact with the tail of MLL [11], is observed to move away from the core of the protein, facilitating MLL binding. Zor and coworkers [26] have shown that the C-terminal helix of KIX is partially disordered. However, melting or unfolding has not been observed on the time scales of the MD simulations of this work. Here, we observe that the α_3_ helix of KIX moves away from the core of the protein with relatively higher amplitude fluctuations, however, still keeping its correlated fluctuations with the N-terminal region of KIX and c-Myb.

### The correlated network of interactions in allostery

**Figure 4:**
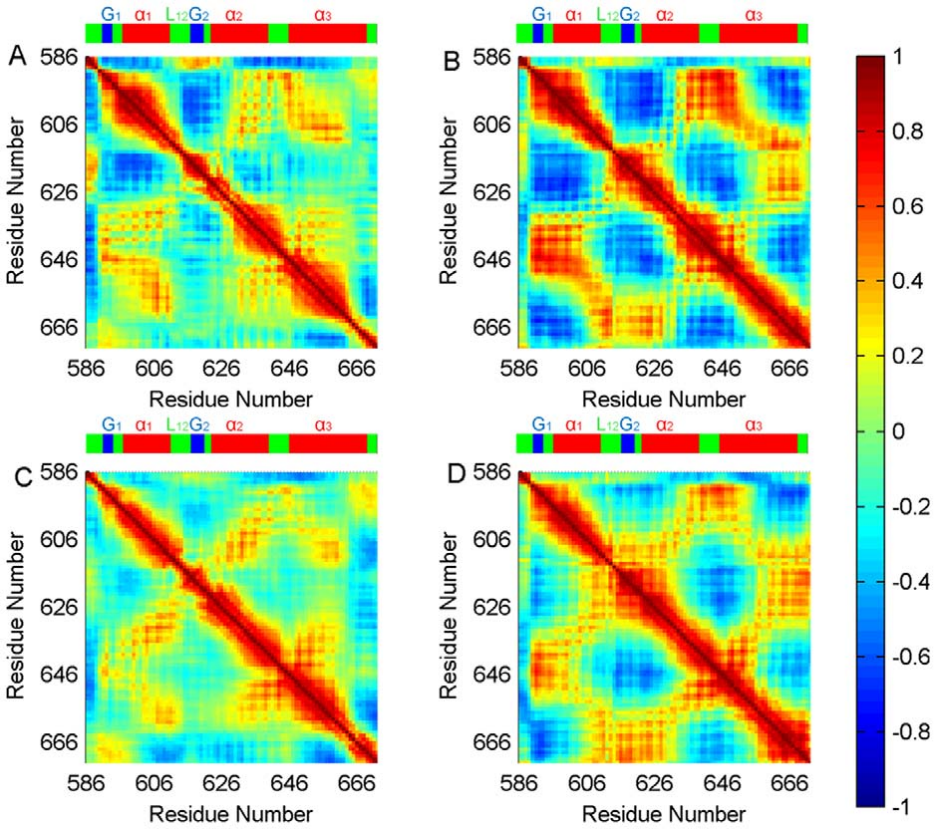
Cross correlations of fluctuations of KIX from different simulations. The cross-correlation maps of KIX from the simulations of: (A) KIX-only, (B) KIX∶MLL, (C) c-Myb∶KIX, (D) c-Myb∶KIX∶MLL. The color scale is represented on the right ranging from red to blue: highly positive correlations are in red, highly negative correlations are in blue. Colors in between relate to the scale among these extremes. The numbers on the axes refer to residue positions. The figure illustrates the strong coupling between the Ala630- Ser642 region and the His592-Pro617 region; Lys606-Leu628 region with His651-Glu666. The cross-correlations of the motions between these regions imply that they play an important role in the propogation of the allosteric effect upon MLL binding to KIX. The color bar on the top of each map stands for the secondary structure (α-helix: Red, Loop: Green, 3_10_-helix: Blue).

The network of correlated fluctuations of the KIX-only structure (Figure 4a) demonstrates that the fluctuations of the Ala630-Ser642 region from the α_2_ helix and the His592-Pro617 region from the G_1_ and α_1_ helix, which contain both the MLL and the c-Myb binding sites, are positively correlated. That can be an indication of the movement of the loop (G_2_ and L_12_) as discussed in the previous section. With MLL binding the correlation increases (Figure 4b), because the L_12_ and G_2_ region moves towards MLL and enhances the interaction network. In addition, the Lys606-Leu628 region, which includes L_12_, G_2_ and the beginning of the α_2_ helix, has stronger correlations with His651-Glu666 in the α_3_ helix in the KIX∶MLL bound form. Expectedly, those regions form a favorable groove for MLL binding. These two regions were previously suggested to be critical for forming a coupled network of interactions through which the propagation of the allosteric effect following MLL binding is transmitted to the c-Myb binding groove [7], [11], [32].

In the case of the c-Myb∶KIX simulation, the correlations of Lys606-Leu628 with His651-Glu666 fade again in comparison to the isolated KIX and KIX∶MLL simulations. Nonetheless, the correlations of the Lys621-Arg646 in the α_3_ helix and the Trp591-Asp616 region of the α_1_ helix along with the G_2_ and L_12_ regions can still be observed as compared to the KIX-only simulation (Figure 4c). Besides, from these correlations it can be inferred that c-Myb binding affects the α_3_ helix with which MLL makes contacts. When the side chains of the C-terminal are investigated, in the case of the KIX and KIX∶MLL binary complex simulations, the side chains of the α_3_ helix point inwards to the MLL binding groove. This could favor the formation of the network of interactions of the α_3_ helix with the rest of the structure. On the other hand, upon c-Myb binding (the c-Myb∶KIX simulation), the side chains of the α_3_ helix point outwards. Thus, it can be deduced that the presence of MLL affects the side chains of the α_3_ helix to strengthen the communication network with the rest of the structure which in turn would allosterically favor c-Myb binding.

When the cross correlations are calculated from the simulation of the ternary structure all correlations (Lys606-Leu628 with His651-Glu666, Lys621-Arg646 with Trp591-Lys621) are emphasized (Figure 4d). Expectedly, the correlations of two regions Lys606-Leu628 and His651-Glu666 of the α_3_ helix are more pronounced for the c-Myb∶KIX∶MLL case. This result is an indication that the two regions (Lys606-Leu628 and His651-Glu666) have roles in allosteric signal transmission. Similar results are obtained in parallel simulations (See Figure S3).

The most compelling conclusion based on the cross correlation analysis of the pseudo-dihedral angles along with the phi and psi angles (Figures S4, S5 and S6), is that the G_2_ and L_12_ region has strong correlations with the C-terminal α_3_ region, with both having a role in the formation of the network of interactions which pre-organize the microenvironment for the MLL binding. In parallel to our findings up to this point, these correlations fade in the cross correlations of the pseudo-dihedral angles as well as in the phi and psi angles calculated over the c-Myb∶KIX binary complex simulation. On the other hand, correlations of the same regions for the c-Myb∶KIX∶MLL complex remain as expected (Figures S4, S5 and S6). Thus, the formation of coupled correlations increases when MLL is present.

### Shift in the ensemble of conformations

**Figure 5:**
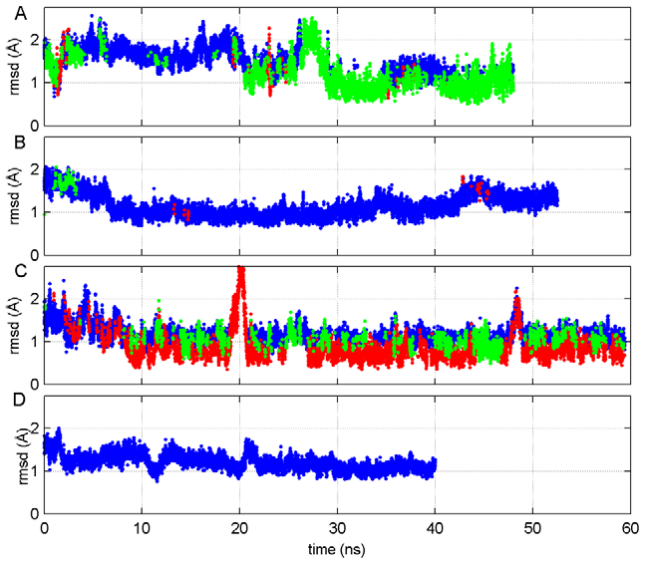
Combinatorial clustering of the KIX ensemble sampled in different simulations. The combinatorial clustering of all KIX conformations sampled in the KIX-only (A), KIX∶MLL (B), c-Myb∶KIX (C) and c-Myb∶KIX∶MLL (D) simulations. Blue, green and red dots represent the members in different clusters. The clustering observed in the figure points to the redistribution of the KIX populations upon c-Myb binding (C). This is because the frequency of occurrence of the KIX conformations is similar to those that KIX has when in the c-Myb∶KIX∶MLL case (blue dots). However, it decreases as compared to KIX (A), KIX∶MLL (B) or the c-Myb∶KIX∶MLL (D) cases.

The KIX, KIX∶MLL, and c-myb∶KIX∶MLL simulations all show similar patterns in the cross-correlation maps, reflecting the outcome of the redistributions of the ensemble of conformations. To further investigate how the ensemble of KIX shifts and what are the prevailing states in the various forms (KIX, KIX∶MLL, c-Myb∶KIX, c-MYb∶KIX∶MLL), we perform clustering over the sampled conformations during the simulations. [33], [34] We follow a combinatorial approach: all the conformations which were created from the four models are provided as the input to the clustering algorithm. [34] Conformations within 2.75 Å RMSD were clustered, leading to three clusters of conformations as indicated by different colors in Figure 5. As can be clearly seen, the conformations from the KIX-only simulations exist in all three clusters (red, blue and green); yet the majority of the conformations distributed between two clusters (blue and green). With MLL binding, the KIX conformations are mainly observed in the blue cluster, which is the cluster of almost all of the KIX bound to both MLL and c-Myb conformations. This clearly indicates that following MLL binding, KIX already assumes the conformational ensemble which it presents when bound to both MLL and c-Myb. In contrast, the conformational ensemble of KIX bound to c-Myb, is distributed among the three clusters, where the conformations of KIX bound to both MLL and c-Myb are not as frequently visited as in the case of KIX bound to MLL (Figure 5). Quantitatively, 58% of the ensemble of isolated KIX conformations belongs to the ensemble of c-Myb∶KIX∶MLL simulation. This value increases to 99% when the ensemble of KIX∶MLL is considered, which implies that the binding of MLL to KIX results in a shift in the ensembles of KIX towards a more favorable state so that the c-Myb∶KIX∶MLL ternary structure can form. On the other hand, the c-Myb∶KIX, which is comprised of the c-Myb∶KIX binary structure, has only 47% resemblance to the ternary complex which is close to the case of the isolated KIX, suggesting that conformations suitable for ternary complex formation are also present in the c-Myb∶KIX case. The most visited cluster in the c-Myb∶KIX ensemble is shown in red. It consists of conformations with the loop region bending outwards. Furthermore, the percentage of the stable conformations (shown by blue dots) in the simulation of the KIX∶MLL binary complex is much higher than in the simulation of isolated KIX, clearly indicating that KIX∶MLL binds to c-Myb with a higher affinity than KIX itself. The results are consistent with those of parallel simulations (See Figure S7).

In light of these findings, it is clear that MLL binding results in a redistribution of the conformational ensembles of KIX such that it favors c-Myb binding.

### The significance of the L12 region: Mutations

According to the correlation maps and the observed motion of loop L_12_ and G_2_ in the Lys606-Leu628 region, we identified this loop region as essential for ternary complex formation with the sequence of events starting from MLL binding to KIX. In the presence of MLL, the loop (L_12_ and G_2_) is bent towards it. On the other hand, upon c-Myb binding to KIX, Met625-Leu628 which resides at the N-terminal of the α_2_ helix is observed to change its conformation in the opposite direction. The significant difference between KIX∶MLL and c-Myb∶KIX in conformations and in correlation maps suggests that these residues could be the key in controlling the bending of the L_12_ and G_2_ regions and thus the allosteric switch. To provide further evidence for the importance of residues in the Met625-Leu628 region for the coupling of the loop motions, *in silico* mutations are performed for selected residues. A change of one residue in that region could interfere with the motion of the loop and block the ternary complex formation.

In order for the Met625-Leu628 region to be more flexible and act like a hinge as in the case of KIX and c-Myb∶KIX simulations, the loop region should bend outward, rather than towards MLL. We selected Glu626 and Leu628 [35] in the KIX∶MLL structure as representative residues and mutated them to Ala. The selection of these particular residues is based on their correlation maps and the fact that the orientation of their side chains displayed considerable changes in the simulations of different models.

The total energies (the kinetic and potential energies of each frame) of E626A and L628A mutants and wild type of KIX∶MLL are presented in Figure S8, using an approach which is similar to that used in the previous *in silico* mutant MD simulation studies [30], [31]. As the figure shows, for both mutants, the total energies remain stable over time. The differences between the models simply arise from the change in the interaction network with the mutations. The RMSF and RMSD of the core region of KIX from the wild type, E626A and L628A mutants of the KIX∶MLL complex, are shown in Figures S9 and S10. None provide any evidence that the mutations destabilize the region. All simulations indicate energetically and conformationally stable ensembles for these two mutations. Several other mutations on the same loop were designed such as P613A, T614A and D622A all of which disrupted the structural and energetic stability of the structure. On the other hand, some mutations, such as P613A did not lead to any change in the cross-correlation maps or in the loop motion, retaining the allosteric relationship between the two binding sites of KIX.

**Figure 6:**
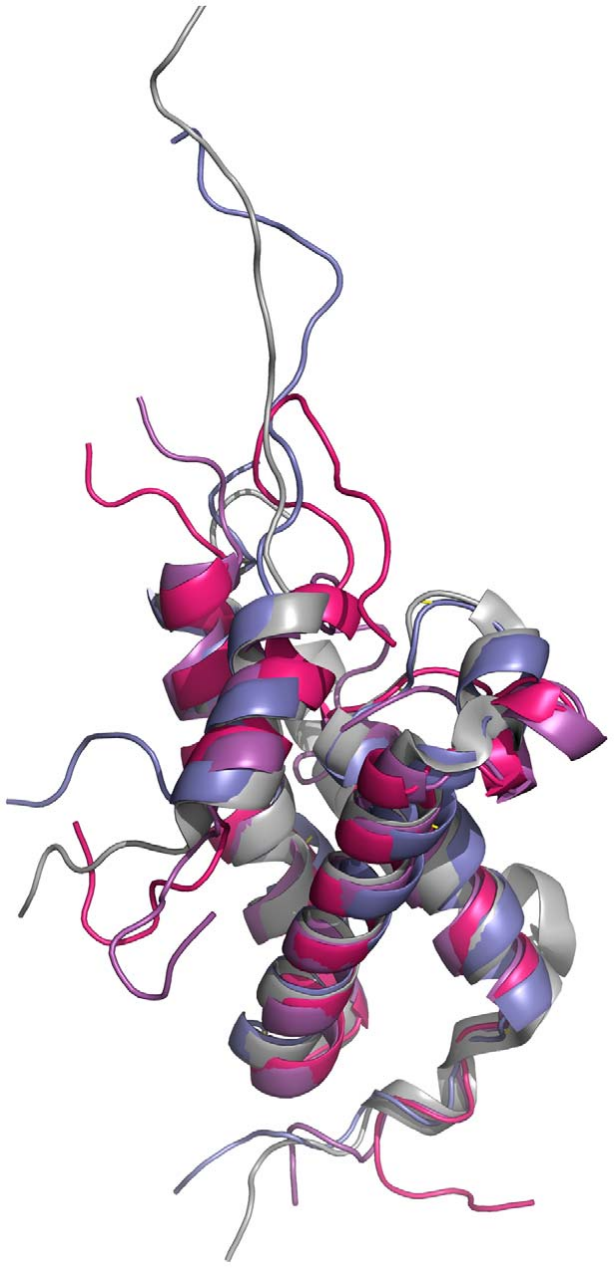
The loop behavior in Glu626Ala and of Leu628Ala mutants. Alignment of the average KIX structure from the simulations of Glu626Ala mutant of KIX∶MLL complex (pink), Leu628Ala mutant of KIX∶MLL complex (purple) and KIX∶MLL structure from the NMR structure (grey) (PDB ID: 2AGH [11]) onto the average structure obtained by the KIX∶MLL wild type simulation (blue) shown using PyMOL [44].

Analysis of the Glu626Ala and Leu628Ala mutant trajectories of KIX∶MLL, revealed that the loop region bends outwards, as in the KIX and c-Myb∶KIX structure (Figure 6). This implies that the loop region interferes with the interacting region of the long MLL tail. This mutation clarifies that the outward movement of the loop hampers the interaction of KIX with MLL's tail, which in turn hampers the allosteric communication network. Since the correlations of Lys606-Leu628 with His651-Glu666 fade in the cross correlation maps of the mutant cases (Figure S11a–d) as compared to KIX∶MLL (Figure 4b), it appears that each mutation distorts the network of interactions of KIX in the KIX∶MLL structure; creates couplings similar to those in the c-Myb∶KIX (Figure 4c); and results in a loop conformation that is open, away from the core of the structure, as in c-Myb∶KIX. In this open conformation no distinct couplings are observed in the allosteric network. In our parallel simulations (Table S.1) we have obtained the similar results (Figures 4, S2 and S11). Thus, our mutations could disfavor the allosteric signaling and decrease the affinity of formation of the ternary complex, which is in agreement with the work of Arai and coworkers [35] in which they mutated Leu628Asp on KIX protein and observed very weak MLL binding.

The present MD simulations provide the dynamics on nanosecond time scale (See Table S1). The results are in agreement with parallel simulations, and as such can be considered as statistically significant in the time window of the simulations. However, this does not exclude any mutation-induced effect on the structure and dynamics that might emerge at longer time scales.

### Conclusions

The allosteric effect upon formation of the MLL∶KIX complex favors c-Myb binding; on the other hand, formation of the c-Myb∶KIX complex does not enhance MLL binding [7], [12]. By simulating the KIX, KIX∶MLL, c-Myb∶KIX and c-Myb∶KIX∶MLL structures, we revealed the allosteric mechanism which explains these opposing effects that have crucial consequences in the activation of Pol II-mediated transcription. Our major conclusion is that the L_12_ and G_2_ loop region plays a key role in the conformation control, acting as a switch: in the KIX-only structure it swings toward the MLL-favored position, pre-organizing the MLL binding site. In contrast, when c-Myb binds to KIX, it swings in the opposite direction. When considering the opposite sequence of events, when MLL binds first, a clear correlation emerges between the fluctuations of the two binding sites, and the loop region. Clustering of the merged trajectories from the KIX, KIX∶MLL, c-Myb∶KIX and c-Myb∶KIX∶MLL simulations reveals that MLL binding redistributes the conformational ensemble of KIX and leads to a more stable ensemble and a higher percentage of population of the favored state, explaining the higher binding affinity of c-Myb to KIX∶MLL compared to KIX only [12]. Further, we observe that the conformation of L_12_ and G_2_ loop region is coupled to the α_3_ helix conformational changes, which further help MLL binding. Our correlation maps clearly demonstrate that the L_12_ and G_2_ loop (Lys606-Leu628) and the α_3_ helix (His651-Glu666) regions are involved in the transmission of the allosteric signal. These correlations with the MLL interaction sites, that is, the allosteric network, fade in the c-Myb∶KIX structure due to the absence of MLL. To further test our conclusions, we performed *in silico* mutations of Glu626 and Leu628 residues in the loop region in the KIX∶MLL structure. Our analysis revealed that these mutations led to the loop swinging away from MLL, similar to the conformation observed for the c-Myb∶KIX. That is, the mutations distorted the KIX structure in a way that would block the allosteric signal transmission of MLL.

Allosteric control has been suggested earlier based on conformational dynamics [36]. Here, our results suggest a mechanism of conformational control in the c-Myb∶KIX∶MLL assembly, and provide an insight which can help to understand how KIX can regulate Pol II-mediated transcription. The key element in these allosteric events is the loop region, which appears to be a pivotal component in the allosteric signal transmission from MLL via KIX to the c-Myb binding site. The detailed analysis of the conformational ensembles provides the mechanistic explanation of how the unidirectional allosteric coupling between the two binding sites of KIX is maintained by a non-binding site conformational switch of the loop.

To this end, the ensemble of conformations with residue fluctuations and their correlations provide a means to study inter-relationships between binding or other functional allosteric events. A dynamic control mechanism of function is essential; and here, through such residue fluctuations and correlations, we observed that loops can play a key role. While here we observed their role in KIX, we believe that a mechanism where loops govern allosteric communication is likely to be general. To this end, experimental studies may test the significance of loops and their role in allosteric control of signal transmission.

## Materials and Methods

### The simulated system

Four models were simulated to shed light on the underlying dynamics and allostery elicited by MLL binding to the KIX domain of CREB-binding protein in the c-Myb∶KIX∶MLL system. The initial structures were based on the NMR structure of c-Myb∶KIX∶MLL (PDB id *2AGH* [11]). The systems simulated are: The isolated structure of the KIX domain of CREB-binding protein (wild type); the isolated structure of KIX domain and MLL binary complex (wild type, E626A mutant and L628A mutant); the isolated structure of c-Myb and KIX binary complex (wild type) and the original ternary complex of c-Myb, KIX domain and MLL. The models are created by removing either MLL or c-Myb or both from the NMR structure prior to solvation and minimization. Chlorine and Sodium ions are added to neutralize the overall charge of the system. The protein was solvated in a TIP3P type [37] octahedral water box. The solvated system went through an energy minimization procedure to remove steric overlaps and unfavorable contacts. In principle, obtaining different models by removing parts of a structure may lead to major conformational changes. The RMSD values of the core region of KIX taken from the equilibrated structures with respect to the NMR structure (PDB id *2AGH* [11]) are summarized in Table 1. As can be seen, the modeled starting structures do not show considerable conformational changes. Comparison of the equilibrated KIX∶c-Myb structure to the c-Myb∶KIX NMR structure (PDB id: 1SB0) [26] displays a smaller RMSD (0.96 Å for the core region) than when compared to the co-crystal c-Myb∶KIX structure in the ternary NMR structure (1.63 Å for the core region) (PDB id: 2AGH) [11] (See Figure S12). Lastly, a summary of the MD simulations is given in Table 1.

**Table 1:** Simulation summary.

Simulated Models	Simulation lengths (ns)	RMSD^a^	Equilibration periods^b^ (ns)
KIX	48	1.3	5
KIX (2^nd^ run)	40	1.4	5
KIX∶MLL	52.5	1.2	15
KIX∶MLL (2^nd^ run)	54.5	1.2	15
KIX∶MLL Glu626Ala	44.5	1.5	5
KIX∶MLL Glu626Ala (2^nd^ run)	21.5	1.4	5
KIX∶MLL Leu628Ala	50	1.4	15
KIX∶MLL Leu628Ala (2^nd^ run)	23	1.6	10
c-Myb∶KIX	60	1.7	7.5
c-Myb∶KIX (2^nd^ run)	20	1.7	4
c-Myb∶KIX∶MLL	40	1.3	4
c-Myb∶KIX∶MLL (2^nd^ run)	48.5	1.3	4

a C-α RMSD of the core region of KIX from equilibrated structures from different simulations with reference to NMR structure (PDB id: 2AGH) [11].

b Equilibration periods are obtained from the RMSD trends.

### Simulation details

MD simulations were carried out using the AMBER package with periodic boundary conditions to mimic the boundary effects and include the solvent effect with a relatively small number of particles [38]–[40]. The *ff03* force field was used [41]. Isobaric periodic boundary conditions are used with isotropic position scaling. The initial velocities of atoms are generated at 10 K with a Maxwellian distribution. Next, the temperature was gradually raised to 300K and maintained. The pressure is kept at 1 bar by the Berendsen weak-coupling approach [42]. A time step of 2 fs was used in the Leapfrog algorithm. Coordinates are recorded every 1 ps. The simulation trajectories were then analyzed for the sampled conformations in all models.

### Details of analysis of trajectories

#### RMSD (Root Mean Square Deviation)

RMSD is the measure of the average distance between the atoms of superimposed structures. In our case, the minimized structure is taken as the reference structure and all other snapshots created by MD simulation are superimposed onto that structure. The ptraj module of AMBER 8.0 [39], [40] is used to calculate the Cα-RMSD values.

#### RMSF (Root Mean Square Fluctuations) and correlations between fluctuations

RMSF is a measure of the deviation of the Cα's of each residue with respect to a reference structure. The reference structure is taken as the average structure calculated over the MD trajectory for the period where the system is observed to be under dynamic equilibrium. For all systems studied the equilibration periods are not included in the calculations of fluctuations. The equilibration point is determined by the RMSD plots. The equilibration times are given in Table 1.

The correlation between residue fluctuations (cross correlations) is calculated as:
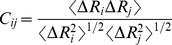
(1)where Δ*R_i_* and Δ*R_j_* are the fluctuations of position vectors of residues i and j, respectively. The position vectors are obtained by aligning each conformation to the minimized structure.

Similarly, the correlation between the fluctuations of the pseudo-dihedral as well as the phi and psi angles are calculates as:
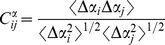
(2)where Δα_i_ and Δα_j_ are the angle fluctuation of residues i and j, respectively. For the virtual bond torsional rotation, the angle is the torsional angle of the virtual bond vector defined between alpha carbon atoms of residues i and j.

The brackets in equations (1) and (2) refer to the ensemble averaging from the equilibrated state to the end of the simulations. The cross correlation values are normalized and range from 1 to −1 with a full positive to negative correlation in residue fluctuations.

#### Clustering

To reduce the conformational space and identify the major conformational states, the conformations sampled during the MD simulations excluding the equilibration periods are clustered by k-means clustering using the kclust module of Multiscale Modeling Tools for Structural Biology (MMTSB) Tool Set [43] having RMSD (in that case the RMSD of functionally important residues are used, see Table S1) of the alpha carbons as the similarity measure [33], [34]. Several thresholds with RMSD from 1.5 Å to 4.0 Å have been tested to identify the ensemble of main conformers. While smaller threshold values led to a larger number of clusters, hardly more than one cluster was obtained with higher threshold values. 2.75 Å has been the optimum threshold value where we could clearly identify and cluster KIX conformations of the four distinct states, in complex with MLL only and c-Myb only, and in complex with both c-Myb and MLL. Nevertheless, a similar trend is still observed with lower and higher threshold values. The clustering is performed over the conformations aligned to the average structure, which is the average of the conformations that reflect the equilibrium dynamics aligned to the minimized structure.

## Supporting Information

Figure S1 **Root Mean Square Fluctuations (RMSF) of KIX from different simulations of parallel runs.** The RMSF of KIX residues from the KIX-only (green), KIX∶MLL (grey), c-Myb∶KIX (blue) and c-Myb∶KIX∶MLL (pink) parallel simulations. The high RMSF values around Phe612∶Lys621 (L_12_ and G_2_) of KIX indicate that this region is the most mobile. Comparing c-Myb∶KIX (green) with KIX (blue) and KIX∶MLL (red) cases, it can be concluded that c-Myb binding rigidifies this region.
(TIF)

Figure S2 **Cross correlations of fluctuations of KIX: A comparison of c-My∶KIX parallel simulations.** The cross-correlation maps of c-Myb and KIX from the two parallel simulations (A and B) of c-Myb∶KIX. The color scale is represented on the right.
(TIF)

Figure S3 **Cross correlations of fluctuations of KIX: A comparison of parallel simulations.** The cross-correlation maps of KIX from the parallel simulations of: (A) KIX-only, (B) KIX∶MLL, (C) c-Myb∶KIX, and (D) c-Myb∶KIX∶MLL. The color scale is represented on the right. The numbers on the axes refer to residue positions. The color bar on the top of each map stands for the secondary structure. (α-helix: Red, Loop: Green, 3_10_-helix: Blue).
(TIF)

Figure S4 **Cross correlations of pseudo-dihedral angles of KIX.** The network of correlated fluctuations of KIX pseudo-dihedral angles from the simulations of: (A) isolated KIX, (B) KIX∶MLL, (C) c-Myb∶KIX, (D) c-Myb∶KIX∶MLL.
(TIF)

Figure S5 **Cross correlations of phi angles of KIX.** The network of correlated fluctuations of KIX phi angles from the simulations of: (A) isolated KIX, (B) KIX∶MLL, (C) c-Myb∶KIX, (D) c-Myb∶KIX∶MLL.
(TIF)

Figure S6 **Cross correlations of psi angles of KIX.** The network of correlated fluctuations of KIX psi angles of KIX from the simulations of: (A) isolated KIX, (B) KIX∶MLL, (C) c-Myb∶KIX, (D) c-Myb∶KIX∶MLL.
(TIF)

Figure S7 **Combinatorial clustering of the KIX ensemble sampled in different simulations of parallel runs.** The combinatorial clustering of all KIX conformations sampled in the KIX-only (A), KIX∶MLL (B), c-Myb∶KIX (C) and c-Myb∶KIX∶MLL (D) simulations. Blue, green and red dots represent the members in different clusters. The clustering observed in the figure points to the redistribution of the KIX populations upon c-Myb binding (C). This is because the frequency of occurrence of the KIX conformations is similar to those that KIX has when in the c-Myb∶KIX∶MLL case (blue dots). However, it decreases as compared to KIX (A), KIX∶MLL (B) or the c-Myb∶KIX∶MLL (D) cases.
(TIF)

Figure S8 **Total energies of KIX∶MLL simulated system (kcal/mol) versus time (ns) from different simulations: Mutant and wild type structures.** Total energies associated with wild type (blue), E626A mutant (green) and L628A (red) of KIX∶MLL simulations are presented. Total energy is the sum of kinetic and potential energies of the whole system including the solvent and the ions.
(TIF)

Figure S9 **Root Mean Square Fluctuations (RMSF) of KIX from different simulations: Mutant and wild type structures.** The RMSF of KIX residues from the Wild Type KIX∶MLL (blue), E626A mutant of KIX∶MLL (red) and L628A mutant of KIX∶MLL (green) simulations.
(TIF)

Figure S10 **Root Mean Square Deviations (RMSD) of KIX from different simulations: Mutant and wild type structures.** The RMSD of KIX residues from the Wild Type KIX∶MLL (blue), E626A mutant of KIX∶MLL (red) and L628A mutant of KIX∶MLL (green) simulations.
(TIF)

Figure S11 **Cross correlations of fluctuations of KIX in mutant simulations.** The network of correlated fluctuations of KIX pseudo-dihedral angles from the simulations of: (A) E626A, 1^st^ run, (B) E626A, 2^nd^ run, (C) L628A, 1^st^ run, (D) L628A, 2^nd^ run.
(TIF)

Figure S12 **Comparison of average structure from c-Myb∶KIX simulation with NMR structures.** 3-D Representation of the average structure from simulation of c-Myb∶KIX (pink) with the NMR structure of c-Myb∶KIX∶MLL ternary complex (PDB id: 2AGH [11]; blue) and c-Myb∶KIX binary complex (PDB id: 1SB0 [26]; green) aligned via PyMOL [44].
(TIF)

Table S1 **Important residues on KIX which are taken as the basis for RMSD comparison in clustering.**
(DOC)

Text S1 **Further information on CBPs.**
(DOC)
