# Supplementary figures and images for: Conformational Control of the Binding of the Transactivation Domain of the MLL Protein and c-Myb to the KIX Domain of CREB
Source: PLoS Comput Biol. 2012 Mar 15;8(3):e1002420. doi: 10.1371/journal.pcbi.1002420 (PMC3305381; doi:10.1371/journal.pcbi.1002420)

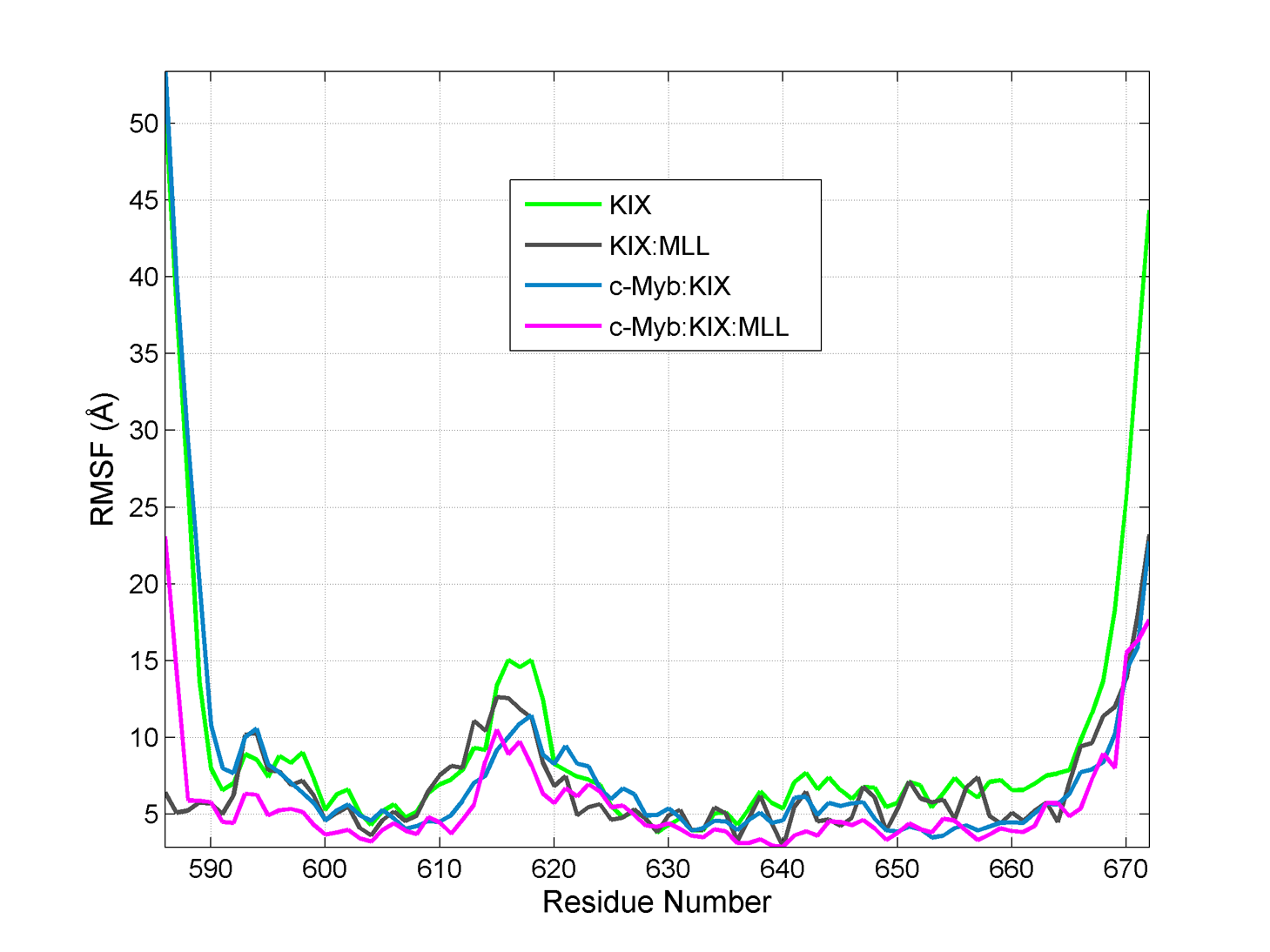

Supplement: Figure S1 — Root Mean Square Fluctuations (RMSF) of KIX from different simulations of parallel runs. The RMSF of KIX residues from the KIX-only (green), KIX∶MLL (grey), c-Myb∶KIX (blue) and c-Myb∶KIX∶MLL (pink) parallel simulations. The high RMSF values around Phe612∶Lys621 (L12 and G2) of KIX indicate that this region is the most mobile. Comparing c-Myb∶KIX (green) with KIX (blue) and KIX∶MLL (red) cases, it can be concluded that c-Myb binding rigidifies this region. (TIF) [file pcbi.1002420.s001.tif]

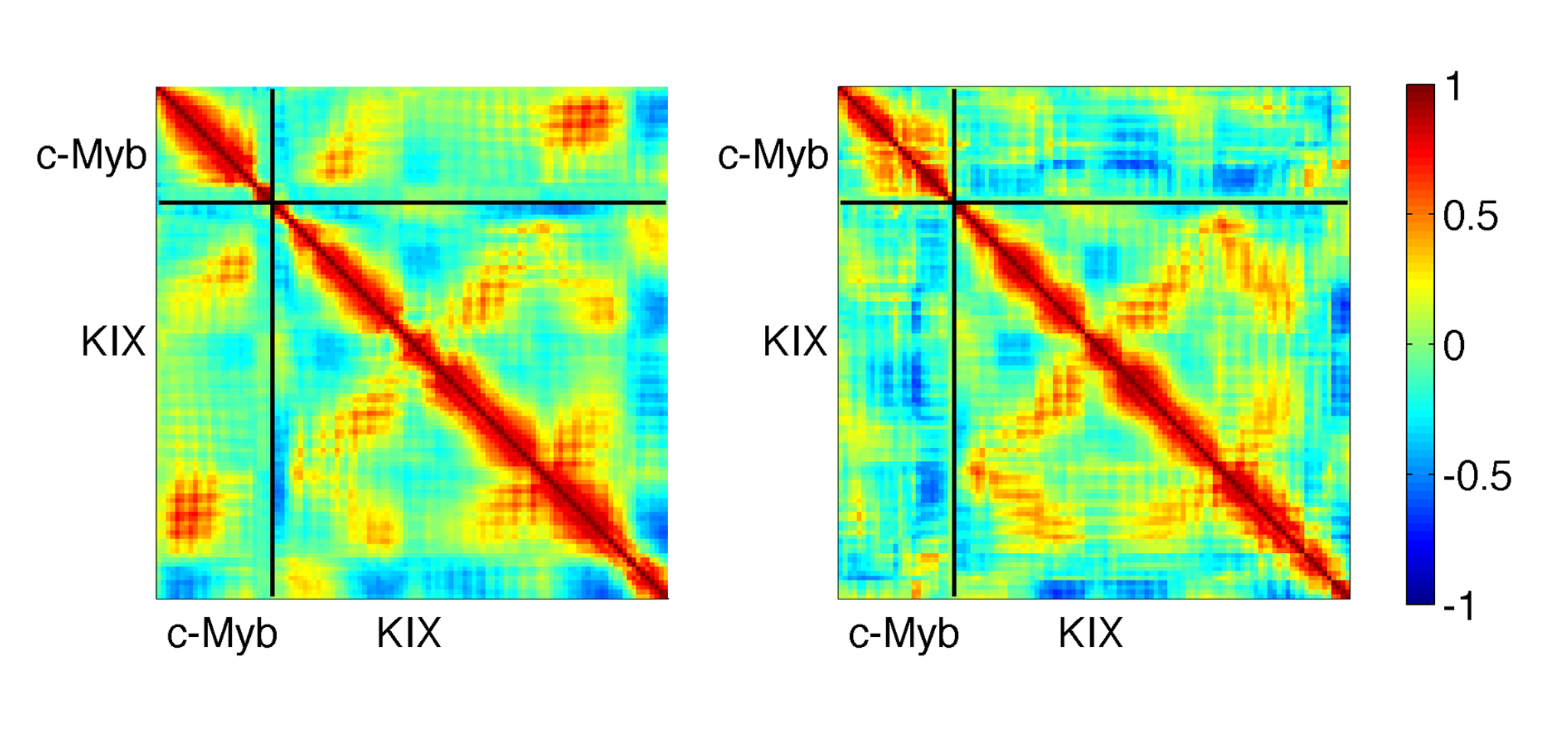

Supplement: Figure S2 — Cross correlations of fluctuations of KIX: A comparison of c-My∶KIX parallel simulations. The cross-correlation maps of c-Myb and KIX from the two parallel simulations (A and B) of c-Myb∶KIX. The color scale is represented on the right. (TIF) [file pcbi.1002420.s002.tif]

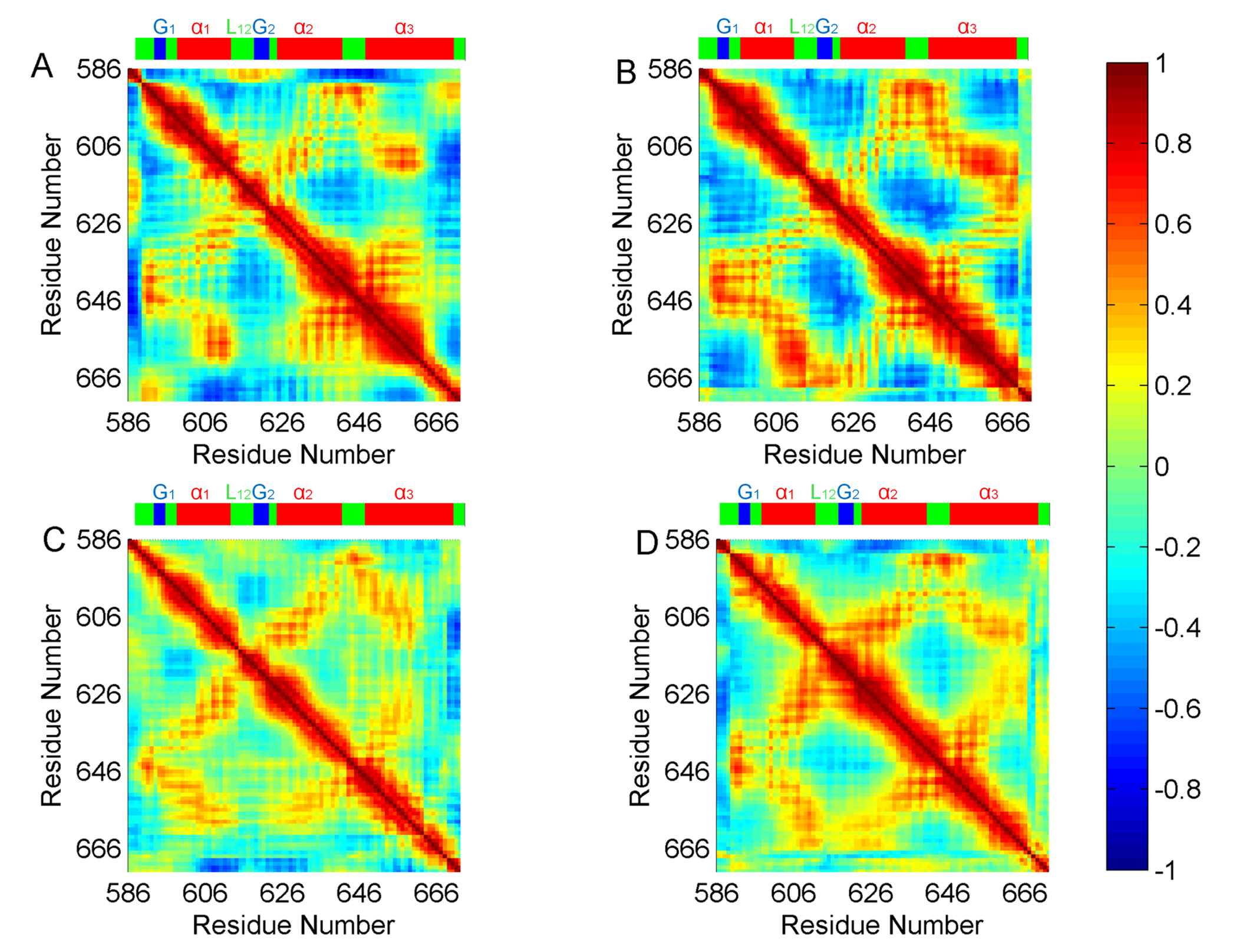

Supplement: Figure S3 — Cross correlations of fluctuations of KIX: A comparison of parallel simulations. The cross-correlation maps of KIX from the parallel simulations of: (A) KIX-only, (B) KIX∶MLL, (C) c-Myb∶KIX, and (D) c-Myb∶KIX∶MLL. The color scale is represented on the right. The numbers on the axes refer to residue positions. The color bar on the top of each map stands for the secondary structure. (α-helix: Red, Loop: Green, 310-helix: Blue). (TIF) [file pcbi.1002420.s003.tif]

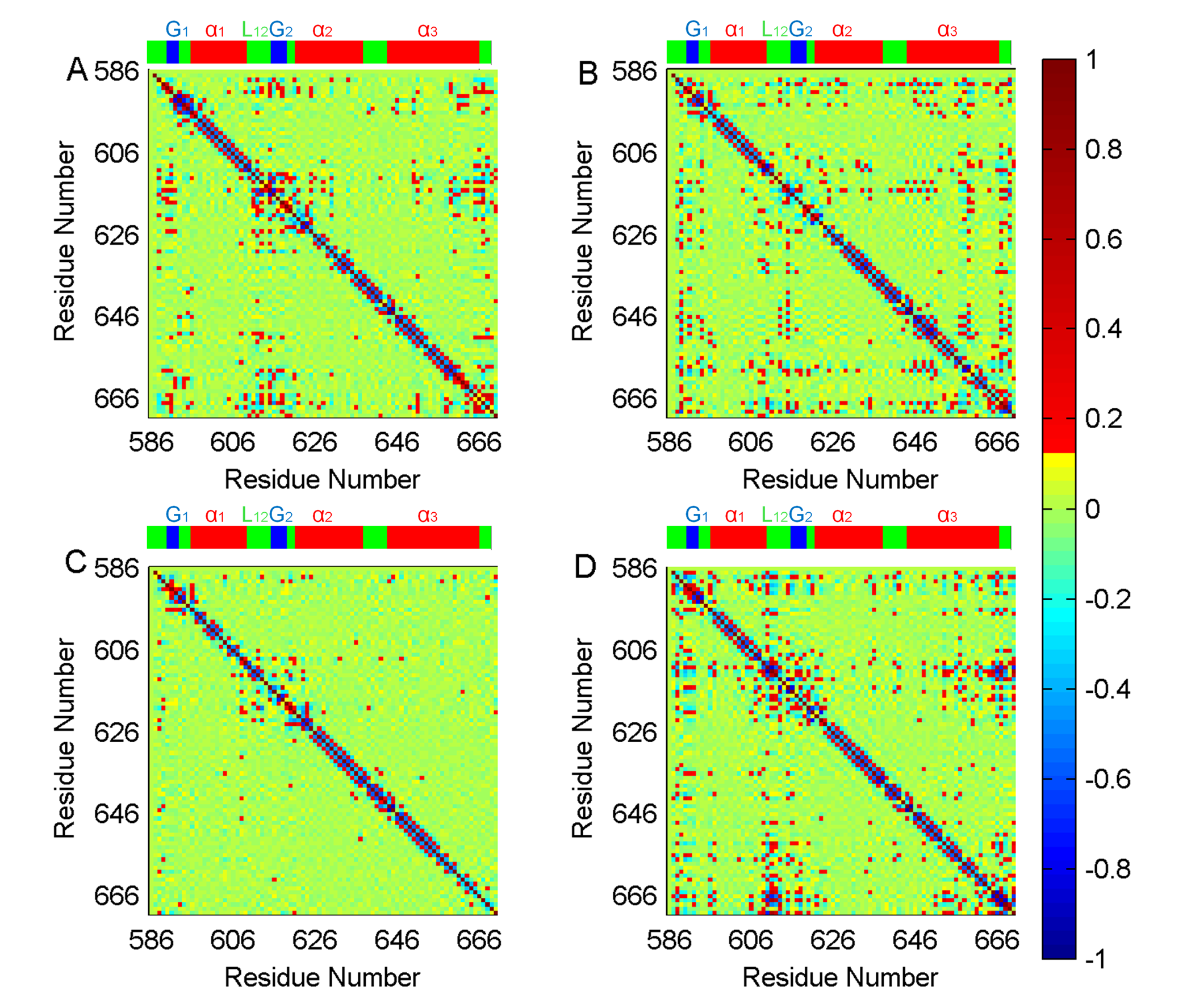

Supplement: Figure S4 — Cross correlations of pseudo-dihedral angles of KIX. The network of correlated fluctuations of KIX pseudo-dihedral angles from the simulations of: (A) isolated KIX, (B) KIX∶MLL, (C) c-Myb∶KIX, (D) c-Myb∶KIX∶MLL. (TIF) [file pcbi.1002420.s004.tif]

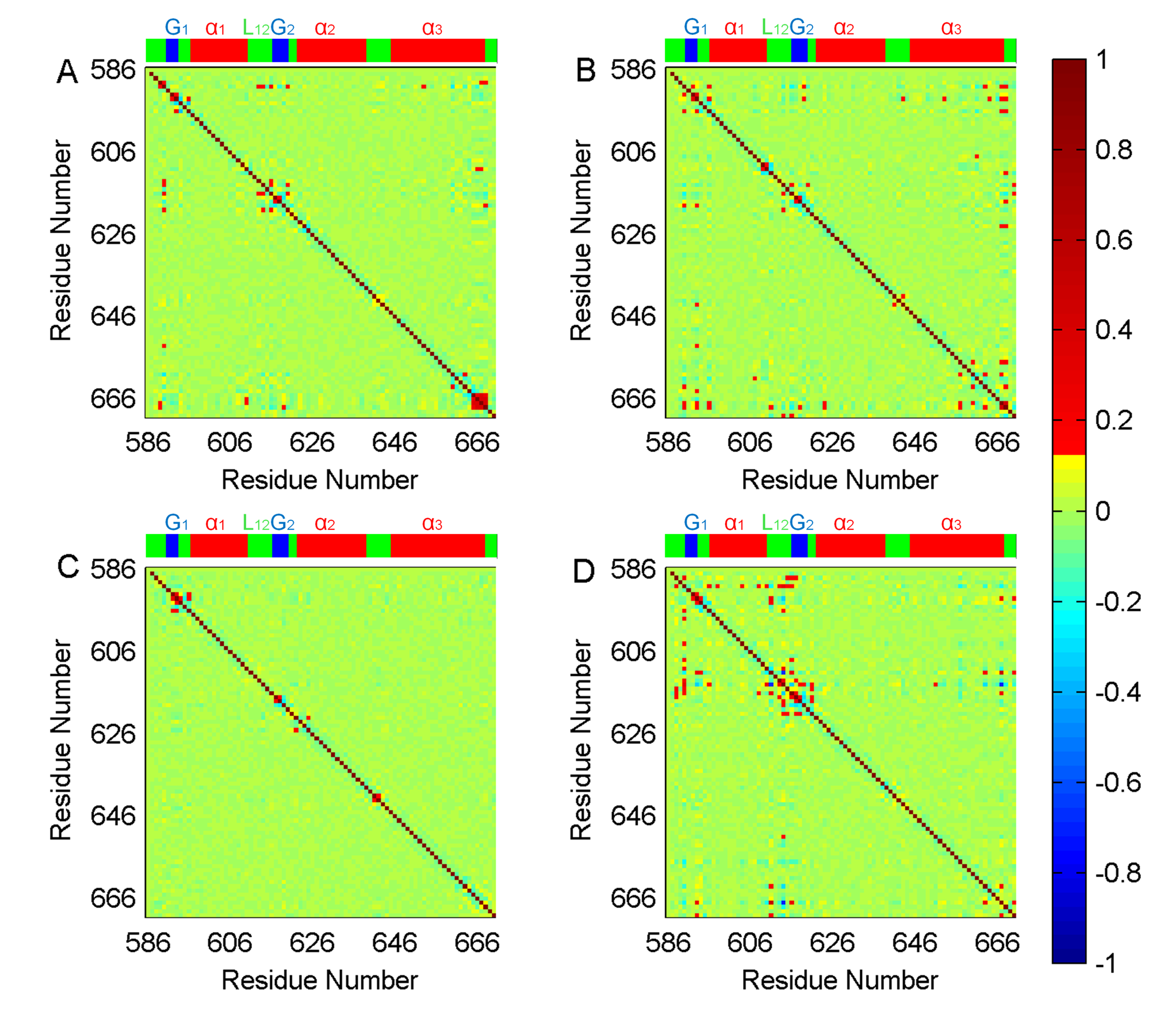

Supplement: Figure S5 — Cross correlations of phi angles of KIX. The network of correlated fluctuations of KIX phi angles from the simulations of: (A) isolated KIX, (B) KIX∶MLL, (C) c-Myb∶KIX, (D) c-Myb∶KIX∶MLL. (TIF) [file pcbi.1002420.s005.tif]

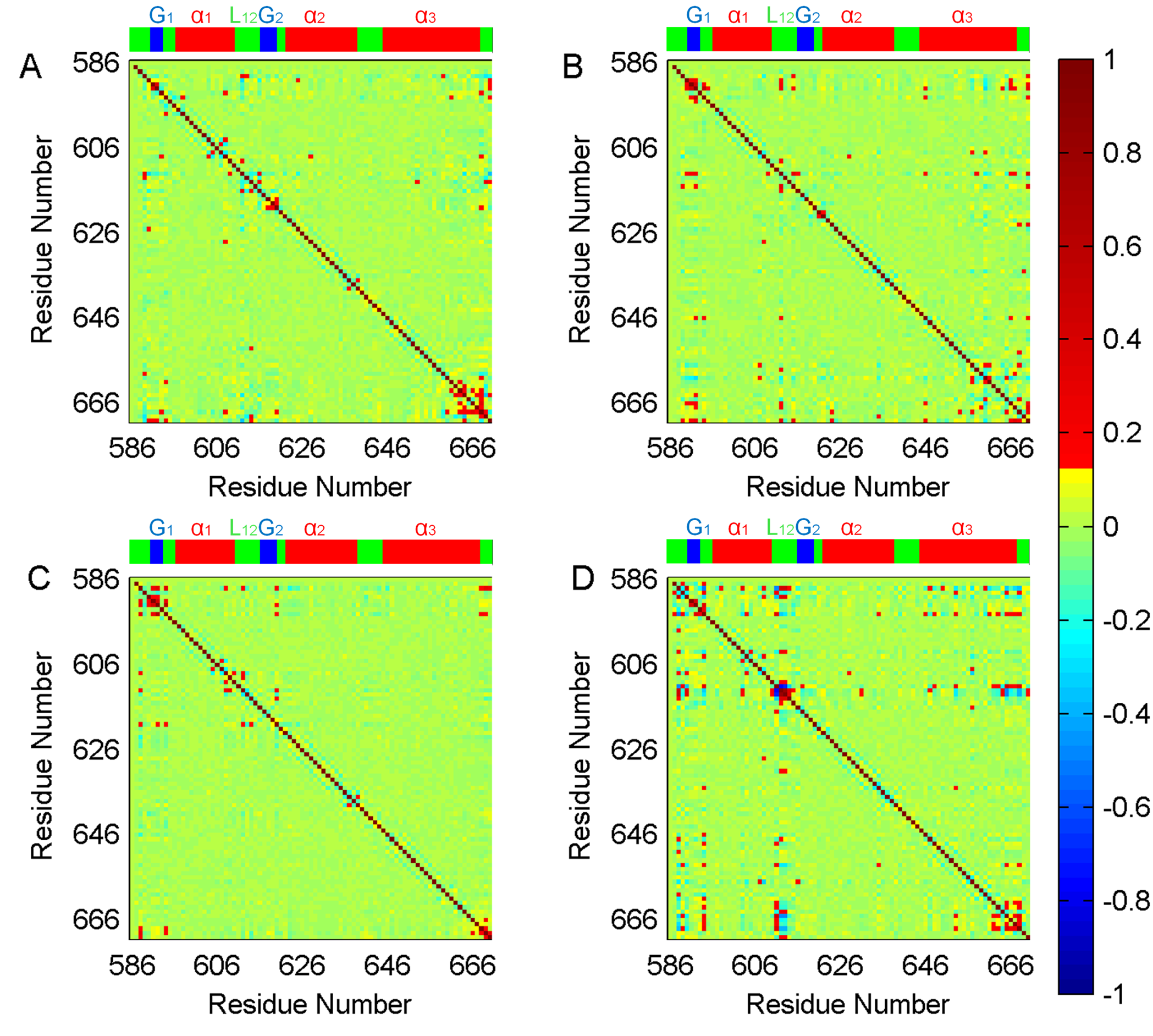

Supplement: Figure S6 — Cross correlations of psi angles of KIX. The network of correlated fluctuations of KIX psi angles of KIX from the simulations of: (A) isolated KIX, (B) KIX∶MLL, (C) c-Myb∶KIX, (D) c-Myb∶KIX∶MLL. (TIF) [file pcbi.1002420.s006.tif]

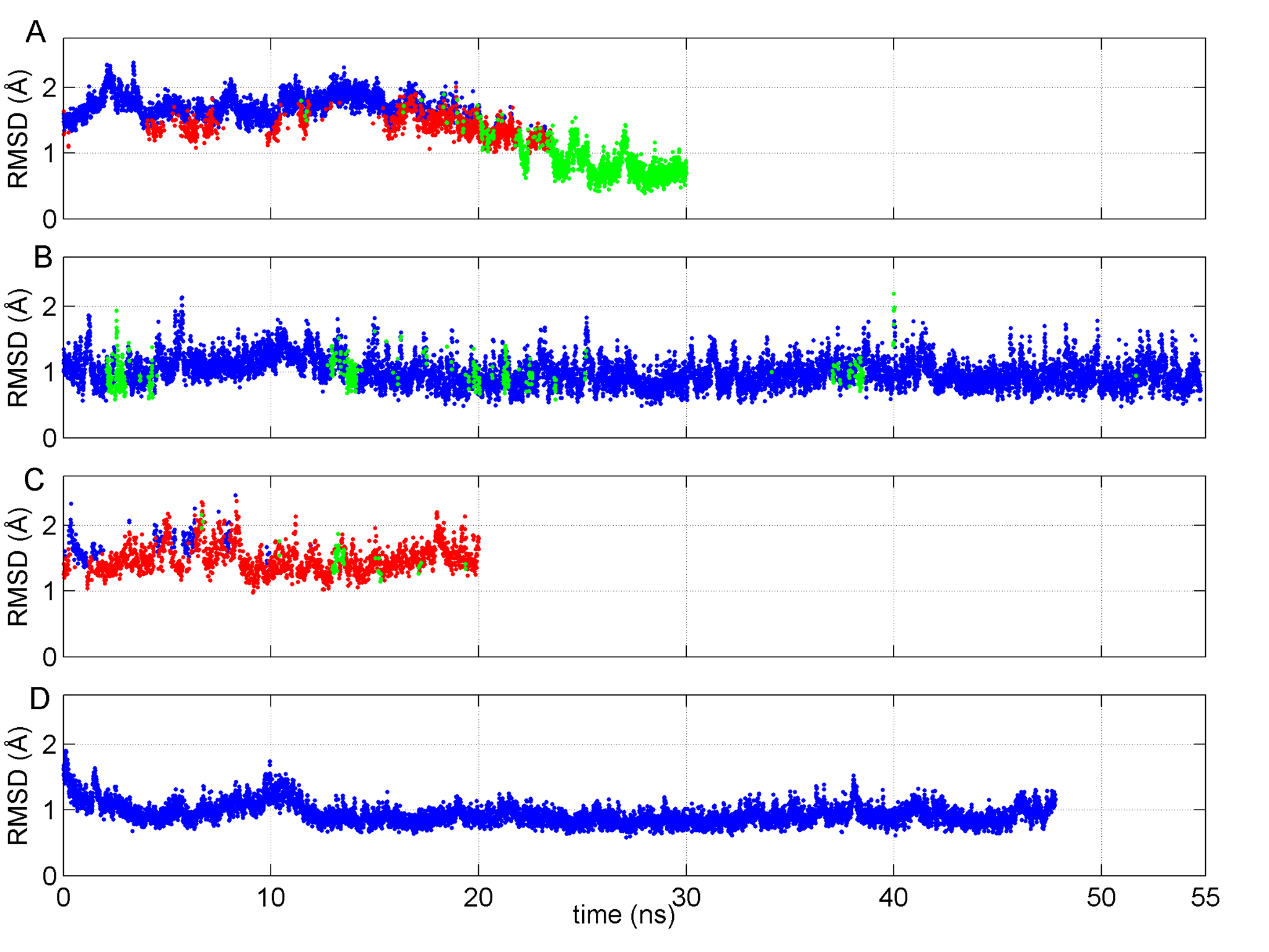

Supplement: Figure S7 — Combinatorial clustering of the KIX ensemble sampled in different simulations of parallel runs. The combinatorial clustering of all KIX conformations sampled in the KIX-only (A), KIX∶MLL (B), c-Myb∶KIX (C) and c-Myb∶KIX∶MLL (D) simulations. Blue, green and red dots represent the members in different clusters. The clustering observed in the figure points to the redistribution of the KIX populations upon c-Myb binding (C). This is because the frequency of occurrence of the KIX conformations is similar to those that KIX has when in the c-Myb∶KIX∶MLL case (blue dots). However, it decreases as compared to KIX (A), KIX∶MLL (B) or the c-Myb∶KIX∶MLL (D) cases. (TIF) [file pcbi.1002420.s007.tif]

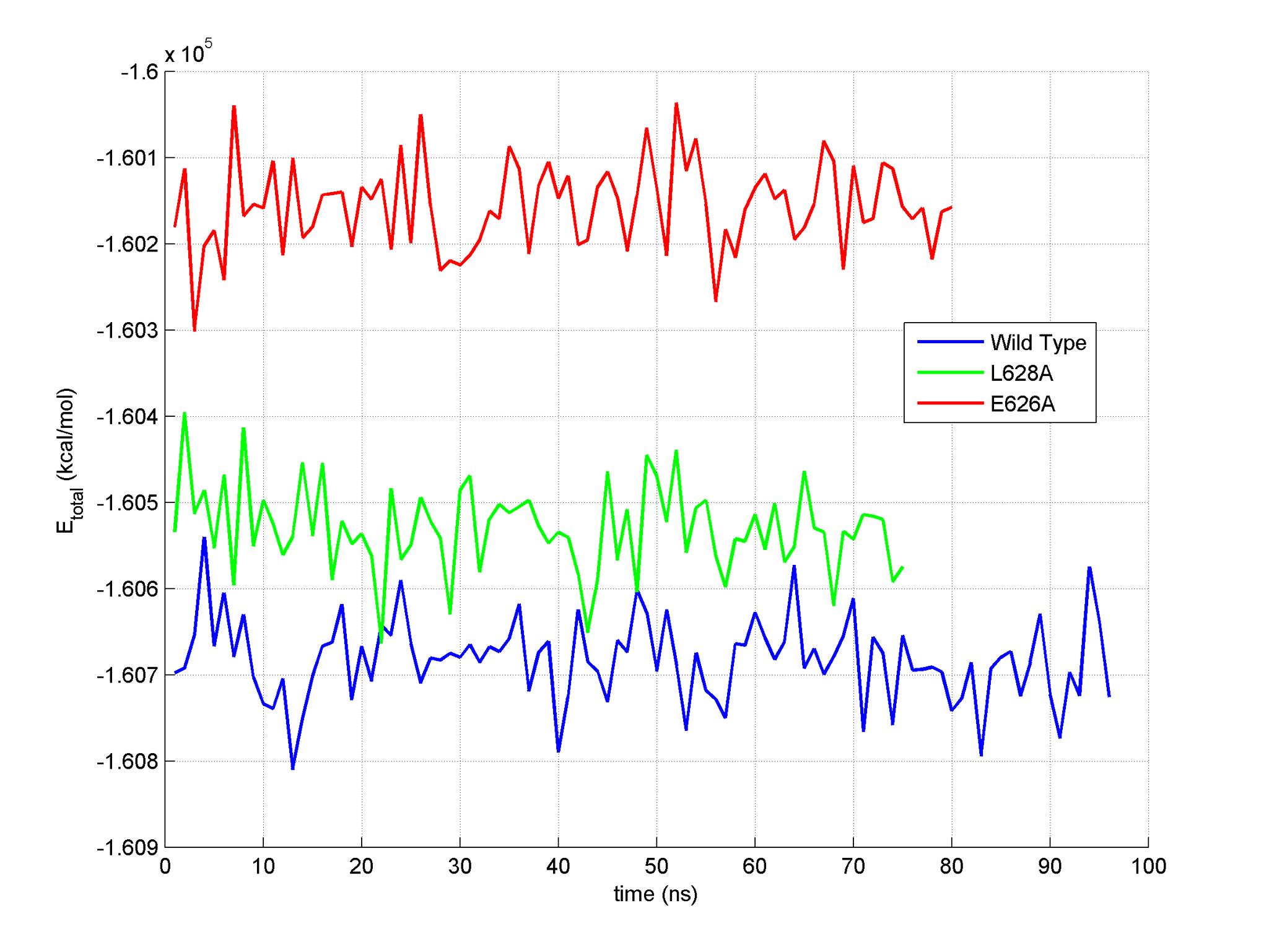

Supplement: Figure S8 — Total energies of KIX∶MLL simulated system (kcal/mol) versus time (ns) from different simulations: Mutant and wild type structures. Total energies associated with wild type (blue), E626A mutant (green) and L628A (red) of KIX∶MLL simulations are presented. Total energy is the sum of kinetic and potential energies of the whole system including the solvent and the ions. (TIF) [file pcbi.1002420.s008.tif]

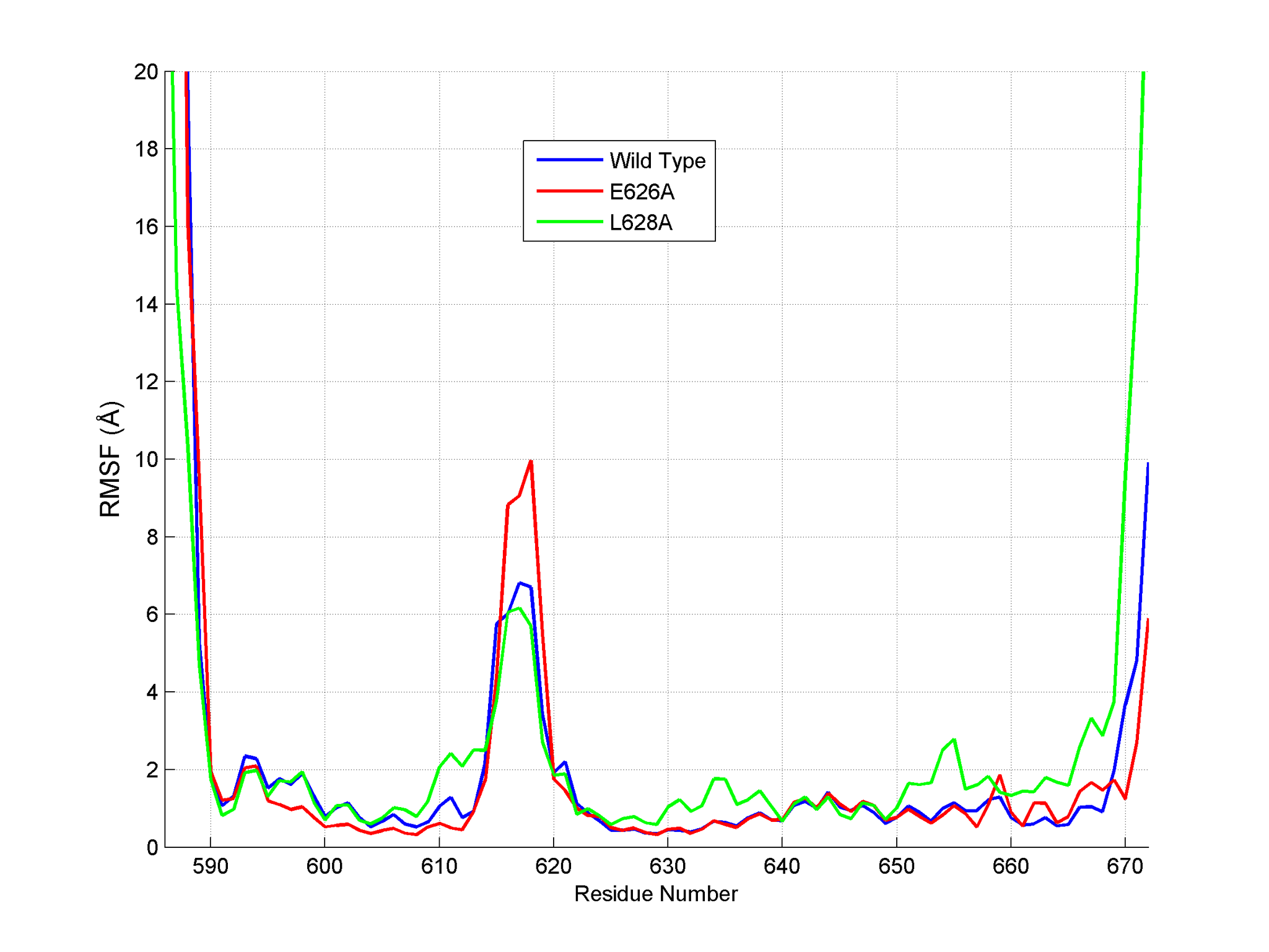

Supplement: Figure S9 — Root Mean Square Fluctuations (RMSF) of KIX from different simulations: Mutant and wild type structures. The RMSF of KIX residues from the Wild Type KIX∶MLL (blue), E626A mutant of KIX∶MLL (red) and L628A mutant of KIX∶MLL (green) simulations. (TIF) [file pcbi.1002420.s009.tif]

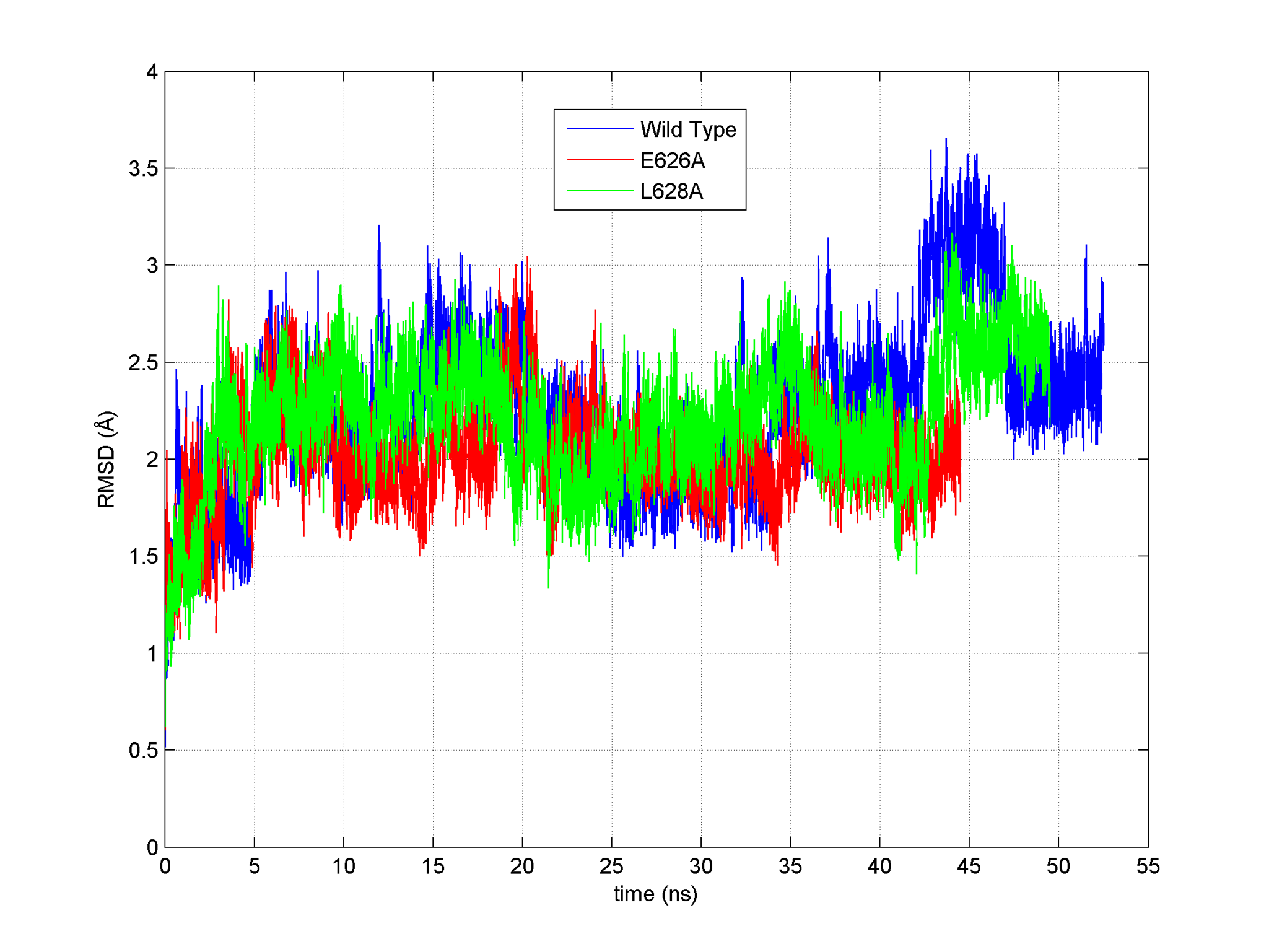

Supplement: Figure S10 — Root Mean Square Deviations (RMSD) of KIX from different simulations: Mutant and wild type structures. The RMSD of KIX residues from the Wild Type KIX∶MLL (blue), E626A mutant of KIX∶MLL (red) and L628A mutant of KIX∶MLL (green) simulations. (TIF) [file pcbi.1002420.s010.tif]

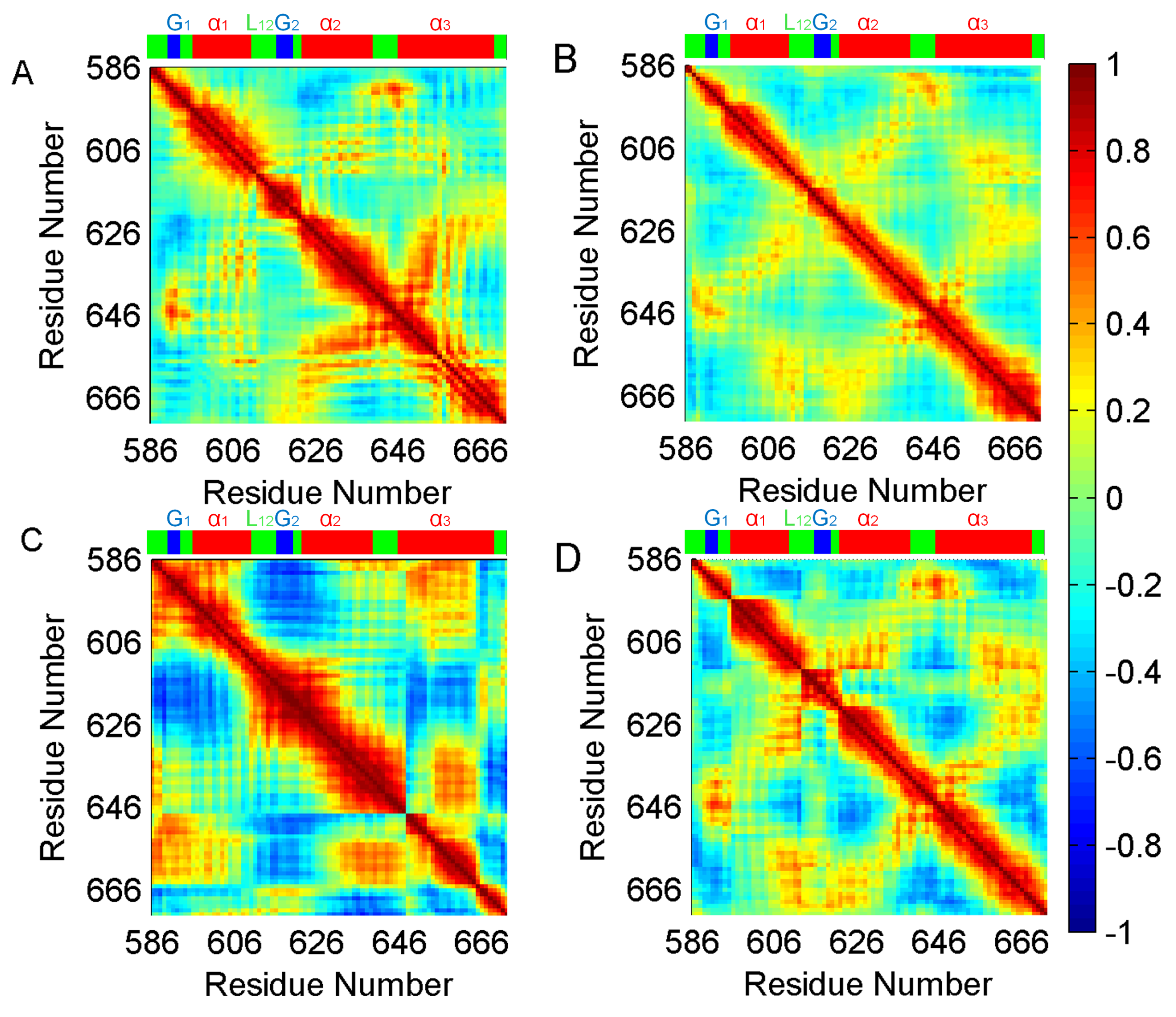

Supplement: Figure S11 — Cross correlations of fluctuations of KIX in mutant simulations. The network of correlated fluctuations of KIX pseudo-dihedral angles from the simulations of: (A) E626A, 1st run, (B) E626A, 2nd run, (C) L628A, 1st run, (D) L628A, 2nd run. (TIF) [file pcbi.1002420.s011.tif]

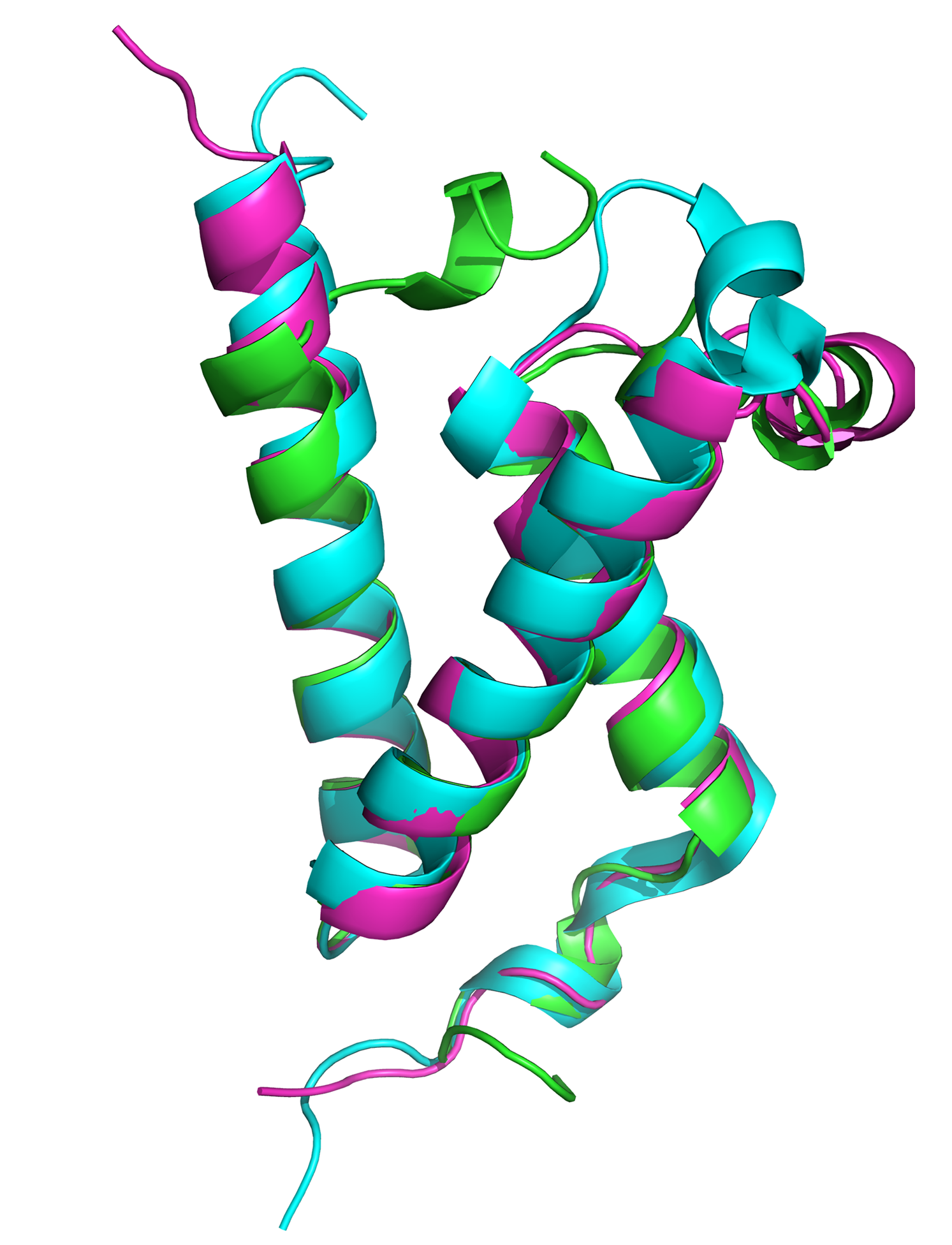

Supplement: Figure S12 — Comparison of average structure from c-Myb∶KIX simulation with NMR structures. 3-D Representation of the average structure from simulation of c-Myb∶KIX (pink) with the NMR structure of c-Myb∶KIX∶MLL ternary complex (PDB id: 2AGH [11]; blue) and c-Myb∶KIX binary complex (PDB id: 1SB0 [26]; green) aligned via PyMOL [44]. (TIF) [file pcbi.1002420.s012.tif]
