# Supplementary material for: Conformational Control of the Binding of the Transactivation Domain of the MLL Protein and c-Myb to the KIX Domain of CREB
Source: PLoS Comput Biol. 2012 Mar 15;8(3):e1002420. doi: 10.1371/journal.pcbi.1002420 (PMC3305381; doi:10.1371/journal.pcbi.1002420)
Supplement: Table S1 — Important residues on KIX which are taken as the basis for RMSD comparison in clustering. (DOC) [file pcbi.1002420.s013.doc]

**Table S1. Important residues on KIX which are taken as the basis for RMSD comparison in clustering.**

| Residues used for RMSD in clustering [7,12] |
| --- |
| Ile611 |
| Phe612 |
| Thr614 |
| Leu620 |
| Lys621 |
| Asp622 |
| Arg623 |
| Arg624 |
| Glu626 |
| Asn627 |
| Tyr650 |
| His651 |
| Ala654 |
| Ile657 |
| Tyr658 |
| Lys659 |
| Ile660 |
