# Supplementary material for: Conformational Control of the Binding of the Transactivation Domain of the MLL Protein and c-Myb to the KIX Domain of CREB
Source: PLoS Comput Biol. 2012 Mar 15;8(3):e1002420. doi: 10.1371/journal.pcbi.1002420 (PMC3305381; doi:10.1371/journal.pcbi.1002420)
Supplement: Text S1 — Further information on CBPs. (DOC) [file pcbi.1002420.s014.doc]

**Text S1. Further information on CBPs**

CBP, which is a transcriptional regulator of RNA polymerase II-mediated transcription, was first characterized as a protein binding to phosphorylated form of CREB in 1993. [17] CBP is a modular transcriptional coactivator which takes part in the regulation of various transcriptional processes. [13,16] CBP is expressed widely and interact with a large number of transcription factors via specific domains. The CBP comprises of 8 domains: an N terminal nuclear receptor binding domain, three cysteine-histidine-rich domains, a bromodomain, two zinc finger motifs and a histone acetyltransferase (HAT) domain. Specifically, the KIX domain of CBP takes part in the integration of signals from numerous signaling pathways in binding to various coactivators. [41] Having various interacting domains, CBP functions as a bridge to transcriptional machinery, a scaffold for the assembly of multiprotein complexes and acetylation-dependent transcriptional regulation. [14,18-20] Being in association with many different molecules such as transcription factors, signaling molecules, hormone receptors etc., CBP is related with many diseases. Mutations of CBP is known to lead Rubenstein-Taybi Syndrome [21,22], certain types of cancers including breast and overian cancers [13], neurodegenerative diseases such as Huntington’s disease, dentatorubral pallidoluysian atrophy, spinal bulbar atrophy, some hematological malignancies including acute myeloid leukemia (AML), chronic myeloid leukemia (CML) and myeloidysplastic syndrome (MDS). [18-20,23] CBPs are also related to cellular growth, differentiation, proliferation and apoptosis. [13-15] More recent findings point out that the dosage of the protein is also essential for regulation of normal differentiation, growth control and homeostasis in human. Right amount of CBP prevents RTS and neurodegenerative diseases. [42] Findings indicate that CBP may also function as a tumor suppressor through direct and indirect ways. [13,15,16,20]
